# Human intestinal beta defensins inhibit viral replication and are diminished in chronic untreated HIV infection

**DOI:** 10.1186/1742-4690-9-S2-P202

**Published:** 2012-09-13

**Authors:** B Corleis, WG Gostic, JA Johnson, DS Kwon

**Affiliations:** 1 Massachusetts General Hospital, Boston, MA, USA

## Background

One of the hallmarks of HIV infection is an early and dramatic depletion of CD4 T cells in the intestinal lamina propria. This loss of T cells is accompanied by intestinal epithelial cell disruption resulting in the translocation of luminal bacterial products, persistent systemic immune activation and resultant disease progression. Beta defensins are cationic antimicrobial polypeptides produced by intestinal epithelial cells and phagocytes that are a part of the innate immune response. They have a pivotal role in maintenance of immune homeostasis in the gut. Although beta defensins have been reported to inhibit bacterial and viral replication in vitro, their role in HIV infection has been incompletely characterized.

## Methods

We investigated anti-viral activity and production of defensins in intestinal mucosal biopsies, stool and plasma from individuals with chronic untreated HIV (chronic progressors), immunologically controlled HIV (elite controllers) and HIV uninfected individuals using ELISA, immunohistochemistry, quantitative PCR and viral inhibition assays.

## Results

Plasma levels of beta defensins were increased in the HIV controller group compared to HIV progressors, whereas levels of beta defensins were significantly decreased in biopsies from chronic progressors. Beta defensin release was induced by HIV in ex vivo cultured intestinal cells. Recombinant beta defensins inhibited HIV replication in vitro.

## Conclusion

Beta defensins are upregulated by HIV and inhibit viral replication in vitro. Chronic progressors, however, had diminished levels of beta defensins in gut biopsies and in the plasma. We propose that beta defensins are induced in HIV infection and have a role in inhibiting viral replication and preventing intestinal epithelial cell disruption. In chronic infection, however, their release is diminished, likely due to epithelial damage in the setting of persistent viremia and mucosal inflammation.

